# Health-Related Quality of Life and Stress-Related Disorders in Patients with Bronchiectasis after Pulmonary Resection

**DOI:** 10.3390/jpm13091310

**Published:** 2023-08-26

**Authors:** Alin Nicola, Cristian Oancea, Paula Irina Barata, Mavrea Adelina, Tudor Mateescu, Diana Manolescu, Felix Bratosin, Roxana Manuela Fericean, Raja Akshay Pingilati, Cristian Paleru

**Affiliations:** 1 Department of Thoracic Surgery, “Victor Babes” University of Medicine and Pharmacy Timisoara, Eftimie Murgu Square 2, 300041 Timisoara, Romania; alinnicola.nicola@gmail.com; 2 Doctoral School, “Victor Babes” University of Medicine and Pharmacy, Eftimie Murgu Square 2, 300041 Timisoara, Romania; tudor.mateescu@umft.ro (T.M.); felix.bratosin@umft.ro (F.B.); manuela.fericean@umft.ro (R.M.F.); 3 Center for Research and Innovation in Precision Medicine of Respiratory Diseases, “Victor Babes” University of Medicine and Pharmacy, Eftimie Murgu Square 2, 300041 Timisoara, Romania; oancea@umft.ro (C.O.); barata.paula@student.uvvg.ro (P.I.B.); 4 Department of Internal Medicine I, Cardiology Clinic, “Victor Babes” University of Medicine and Pharmacy Timisoara, Eftimie Murgu Square 2, 300041 Timisoara, Romania; 5 Department of General Surgery, “Victor Babes” University of Medicine and Pharmacy, Eftimie Murgu Square 2, 300041 Timisoara, Romania; 6 Department of Radiology, “Victor Babes” University of Medicine and Pharmacy, Eftimie Murgu Square 2, 300041 Timisoara, Romania; dmanolescu@umft.ro; 7 Department of Infectious Diseases, “Victor Babes” University of Medicine and Pharmacy, Eftimie Murgu Square 2, 300041 Timisoara, Romania; 8 Malla Reddy Institute of Medical Sciences, Suraram Main Road 138, Hyderabad 500055, India; rjakshay48@gmail.com; 9 Department of Thoracic Surgery, “Carol Davila” University of Medicine and Pharmacy, Bulevardul Eroii Sanitari 8, 050474 Bucuresti, Romania; cristian.paleru@gmail.com

**Keywords:** HRQOL, life quality, bronchiectasis, thoracic surgery

## Abstract

This multicenter, cross-sectional study investigates the potential correlation between the development of bronchiectasis after lung resection surgery and the health-related quality of life (HRQoL) of the patients. The study aims to provide new insights into the long-term outcomes of patients post-lung resection surgery. The study includes adult patients who underwent lung resection surgery for suspicious lung nodules and developed bronchiectasis within a follow-up period of six months. Bronchiectasis was confirmed by high-resolution computed tomography scans. The patient’s health-related quality of life (HRQoL), anxiety, depression, and stress-related disorders were assessed using WHOQOL-BREF, SF-36, HADS, and PSS-10 questionnaires. Out of the 135 patients included in the study, 44 developed bronchiectasis after lung resection surgery. No statistically significant differences were observed between the groups in terms of demographics and medical history. Patients with bronchiectasis demonstrated a lower overall health status, increased deterioration of respiratory symptoms, lower physical activity levels, lower quality of life scores, and experienced more severe anxiety symptoms. Additionally, patients in this group also perceived higher levels of stress; although, the correlation with physical functioning was contradictory. The development of bronchiectasis post-lung resection surgery was associated with poorer quality of life, increased respiratory symptoms, higher anxiety levels, and increased perception of stress. While the correlation between bronchiectasis and HRQoL was statistically significant, the contradictory correlations with stress and physical functioning call for further research. This study underscores the importance of ongoing patient monitoring and the detailed evaluation of respiratory function following lung resection surgery for lung nodules, especially among those who develop bronchiectasis.

## 1. Introduction

Bronchiectasis is a chronic pulmonary condition, typically characterized by a cycle of inflammation and infection, leading to a progressive deterioration of lung architecture, manifested as permanently dilated and damaged bronchi [1,2]. This structural alteration compromises the ability of bronchi to clear mucous, providing a favorable environment for recurrent bacterial infections, inflammation, and further lung damage. Bronchiectasis is often associated with serious co-morbidities, significantly impacting the quality of life of affected individuals [3,4].

The development of bronchiectasis is often a result of a complex interplay of etiological factors [5,6]. Among these, one crucial and understated contributor is the role of surgical interventions, particularly pulmonary resections [7,8,9]. These resections, often performed in the treatment of conditions, such as lung cancer or severe emphysema, can emerge as a significant risk factor for post-operative bronchiectasis [10]. The intricacies of this process go beyond lung remodeling and impaired mucociliary clearance. Surgical procedures, particularly those necessitating extensive lung tissue removal, may lead to an imbalance in lung pressure dynamics and disrupt normal airflow. Additionally, post-operative changes, including scarring and fibrosis, can contribute to structural changes predisposing to bronchiectasis. Moreover, post-operative states can lead to weakened immunity and an increased susceptibility to infections, further fueling the pathogenesis of bronchiectasis [11,12,13].

The health-related quality of life (HRQoL) in patients with bronchiectasis is substantially reduced due to persistent symptoms, such as chronic cough, fatigue, dyspnea, and frequent exacerbations [14,15]. Moreover, the continual need for treatments, including antibiotics and physiotherapy, further impinges on patients’ everyday activities, negatively affecting their HRQoL. Several studies have explored this aspect, highlighting the necessity of comprehensive disease management to improve HRQoL [16,17].

Despite the considerable physical burden, the psychological aspects of bronchiectasis, particularly stress-related disorders, have been less explored. The existing literature highlights the significant role of stress and anxiety in patients with chronic respiratory conditions, primarily by worsening their perception of symptoms and impairing adherence to treatments [18]. It can be hypothesized that these psychological stressors may also be prevalent and detrimental in patients with bronchiectasis, particularly those having undergone pulmonary resections. In view of these considerations, there is a clear need to better understand the impact of bronchiectasis post-pulmonary resections on patients’ HRQoL and psychological well-being. The extent to which this condition influences stress-related disorders remains largely under-investigated.

It was hypothesized that bronchiectasis post-pulmonary resections significantly reduce HRQoL and exacerbate stress-related disorders, thereby necessitating comprehensive intervention approaches. Consequently, the primary objectives of this study are to investigate the health-related quality of life in patients with bronchiectasis after a pulmonary resection, and to evaluate the impact of stress-related disorders in this patient group.

## 2. Materials and Methods

### 2.1. Study Design and Ethical Considerations

The current study was designed as a multi-centric cross-sectional study conducted over a period of 6 months from October 2022 to April 2023. The research protocol was approved by the institutional review boards of the institutions Victor Babes Hospital for Infectious Diseases and Pneumology from Timisoara, Romania, and the National Institute of Pneumology Marius Nasta from Bucharest, Romania. All participants provided written informed consent prior to participation, and all procedures complied with the Declaration of Helsinki.

### 2.2. Study Participants and Inclusion and Exclusion Criteria

Patients who were evaluated at the sixth-month appointment were included in the current study if they satisfied the inclusion criteria. The participant inclusion for this study was based on several predefined criteria. Firstly, we considered only adult patients aged 18 years old and above. To ensure accuracy and consistency, the diagnosis of bronchiectasis was confirmed by high-resolution computed tomography (HRCT) scans, in accordance with the established guidelines [19]. These patients underwent pulmonary resection surgery and had their follow-up appointment between October 2022 and April 2023. We specifically looked for patients who developed bronchiectasis post-lung resection surgery for suspicious lung nodules, as identified during their 6-month follow-up. All participants were affiliated with either of the study centers: Victor Babes Hospital for Infectious Diseases and Pneumology from Timisoara, Romania, or the National Institute of Pneumology Marius Nasta from Bucharest, Romania. It was necessary that the participants were capable of understanding and responding to the questionnaires associated with health-related quality of life (HRQoL) and stress-related disorders. The final and critical inclusion factor was the patient’s willingness to participate in the study, indicated by their provision of written informed consent.

The first exclusion criterion was for patients under the age of 18 years old. Patients who underwent a lung resection intervention for pulmonary tuberculosis, pulmonary abscess, congenital lung malformations, fungal infections, emphysema and bullae, or other conditions that necessitated a lung resection, besides a benign lung nodule, were excluded. Another exclusion criterion was when the resected pulmonary lobe that involved the suspicious lung nodule appeared as malignant in the pathology examination. Patients with pre-existing bronchiectasis before the pulmonary resection surgery were also excluded, to avoid conflating bronchiectasis caused by pathologies, such as COPD, with bronchiectasis developing after the lung resection. Patients diagnosed with cancer were not considered for evaluation and they were not present in the current database.

For the control group, we included patients who had undergone lung resection surgery but did not develop bronchiectasis post-surgery. These patients also needed to satisfactorily complete the same HRQOL and stress-related disorder questionnaires as our primary participant group. Further exclusion criteria involved patients with other significant comorbidities that might potentially impact the HRQOL and stress-related disorder assessments. These comorbidities included uncontrolled diabetes, cardiovascular disease, active malignancies, or other severe respiratory disorders, such as cystic fibrosis, which had a high preoperative risk and contraindicated surgery.

For both groups, patients with cognitive impairments or mental health disorders that could interfere with their ability to understand or respond accurately to the study questionnaires were also excluded. We did not include patients who were unable to provide written informed consent for the study, or those with incomplete medical data or those who failed to satisfactorily complete the questionnaires. Finally, patients who declined to participate in the study were also excluded. These exclusion criteria were applied to ensure that all study participants were able to provide reliable responses to our HRQOL and stress-related disorder questionnaires.

**Figure 1:**
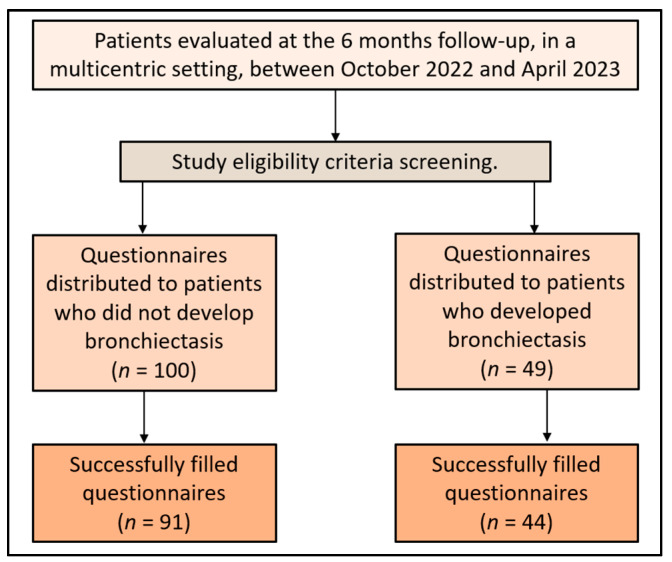
Study flowchart.

The determination of the sample size was conducted using the convenience sampling approach. The ideal sample size was projected for 97 participants, with a 5% margin of error and 95% confidence level. The set threshold for statistical significance was *p* < 0.05. For a type-I error rate of 5%, the statistical power was computed to be 80%. A total of 49 patients with bronchiectasis agreed to participate in the study during the surveying period. The completion of the surveys was facilitated online with the assistance of the study’s affiliated physicians. A total of 45 questionnaires were satisfactorily filled, and after excluding those with missing medical data, 44 were included in the final analysis. For the control group, 100 patients who did not develop bronchiectasis 6 months after the intervention were asked to fill out the same questionnaires. The final analysis included 91 complete answers from patients who did not develop bronchiectasis, as presented in Figure 1.

### 2.3. Questionnaires

A total of four standardized questionnaires were considered for use in the current study, including WHOQOL-BREF, SF-36, HADS, and PSS-10. The World Health Organization Quality of Life-BREF (WHOQOL-BREF) is a self-report questionnaire designed to assess the individual’s perception of their position in life, in the context of their culture and value systems, and in relation to their goals, expectations, standards, and concerns [20]. It includes four domains: physical health, psychological health, social relationships, and environment. Each item is rated on a 5-point Likert scale where 1 indicates low, negative perceptions and 5 indicates high, positive perceptions. Domain scores are scaled in a positive direction where higher scores denote a higher quality of life. The instrument contains two individual items related to an individual’s overall perception of quality of life and health, along with 24 items covering the aforementioned domains. WHOQOL-BREF is particularly notable for its international validation and its comprehensive representation of the health-related quality of life construct.

The 36-Item Short Form Health Survey (SF-36) [21] is a commonly used, patient-reported survey that investigates the health status across eight domains: vitality, physical functioning, bodily pain, general health perceptions, physical role functioning, emotional role functioning, social role functioning, and mental health. The instrument not only provides an overall view of a patient’s health status but also allows for the evaluation of specific aspects of health and well-being, thus offering a broad perspective on the health-related quality of life. Each scale is directly transformed into a 0–100 scale on the assumption that each question carries equal weight. The lower the score the higher the disability, the higher the score the lower the disability, i.e., a score of zero is equivalent to maximum disability and a score of 100 is equivalent to no disability.

The Hospital Anxiety and Depression Scale (HADS) is a widely used self-assessment scale that helps identify and quantify the twin states of anxiety and depression in a medical outpatient setting [22]. Comprising 14 items, it includes two subscales of seven items each that specifically measure anxiety (HADS-A) and depression (HADS-D). Each item on the questionnaire is scored from 0–3 and this means that a person can score between 0 and 21 for either anxiety or depression. A score of 8–10 is suggestive of the presence of the respective state and a score of 11 or more is almost always indicative of such a state. The scale is designed to avoid reliance on aspects of these conditions that are also common to physical illness, making it particularly suited for a population of patients with physical health conditions, such as those in our study.

Lastly, the Perceived Stress Scale (PSS-10) is the most commonly used tool for measuring the perception of stress [23]. It gauges the degree to which situations in an individual’s life are appraised as stressful during the last month. The items in the PSS-10 ask about feelings and thoughts during the last month and how often one found situations to be unpredictable, uncontrollable, or overloaded. PSS-10 scores are obtained by reversing the responses (e.g., 0 = 4, 1 = 3, 2 = 2, 3 = 1, and 4 = 0) to the four positively stated items (items 4, 5, 7, and 8) and then summing across all scale items. The items are designed to monitor how unpredictable, uncontrollable, and overloaded respondents find their lives. The scale also includes a number of direct queries about current levels of experienced stress. The PSS-10 is not a diagnostic instrument; therefore, there are no cut-off scores. A higher score indicates a higher level of perceived stress. Overall, this scale is an effective tool for measuring whether or how the occurrence and management of stressful events are associated with health and disease, making it an ideal fit for our research.

### 2.4. Statistical Analysis

The data were analyzed using the Statistical Package for the Social Sciences (SPSS) version 26 (IBM Corp., Armonk, NY, USA). Descriptive statistics were used to summarize the demographic characteristics of the study sample, expressed as the mean ± standard deviation for continuous variables and frequencies (percentage) for categorical variables. Independent *t*-tests were used to compare the means of continuous variables, such as age, WHOQOL-BREF, SF-36, HADS, and PSS-10 scores, between cases and controls. The chi-squared test was used for the comparison of categorical variables between the two groups. Effect sizes were calculated using Cohen’s d to determine the magnitude of the differences between the groups. A Cohen’s d of 0.2 was considered a ‘small’ effect size, 0.5 represented a ‘medium’ effect size, and 0.8 a ‘large’ effect size. The correlation between the different health-related quality of life parameters and perceived stress was assessed using Pearson’s correlation coefficient. All tests were two-tailed, and a *p*-value of less than 0.05 was considered statistically significant. The data are presented as mean ± standard deviation (SD) or numbers (percentages).

## 3. Results

### 3.1. Patients’ Demographics and Medical History

The patients’ demographics and medical history for both cases and controls are described in Table 1. There were 44 cases who developed bronchiectasis after lung resection and 91 controls who did not develop bronchiectasis postoperatively. The average age of the patients in the case group was 56.2 years old, while in the control group it was 55.6 years old (*p* = 0.656), indicating a similar age distribution among both the groups. The gender distribution between the cases and controls did not differ significantly either, with 65.9% of the cases and 70.3% of the controls being men (*p* = 0.603). The area of residence, whether urban or rural, was also comparable between the two groups (47.7% of cases and 46.2% of controls resided in urban areas, *p* = 0.863).

Smoking status was assessed in both groups, with 38.6% of cases and 33.0% of controls identified as smokers (*p* = 0.516). Similarly, there was no significant difference in the median pack-year smoking history between the two groups (24.5 vs. 21.0, *p* = 0.219). In terms of exposure to respiratory hazards, 20.5% of the cases and 15.4% of the controls had such exposure in their history (*p* = 0.462). The prevalence of obesity was higher in cases (36.4%) than in controls (24.2%); however, this difference did not reach statistical significance (*p* = 0.139). Lastly, regarding the Charlson comorbidity index (CCI), 22.7% of the cases and 17.6% of the controls had a CCI higher than 3, indicating a high burden of comorbidity; however, this difference was not statistically significant.

### 3.2. Clinical Characteristics and Investigations

Table 2 shows the clinical characteristics and investigations in the study cohort. The mean forced expiratory volume in the first second (FEV1) percent predicted was slightly lower in cases (80.3%) compared to controls (81.8%); however, this difference was not statistically significant. The mean ejection fraction (EF) was also similar between the two groups, with cases and controls showing 57.0% and 56.4%, respectively, and no significant difference was found. All patients, both cases and controls, had benign histologies confirmed after resection surgery. In terms of the lung localization, the left lung was involved in 43.2% of the cases and 41.8% of the controls, while the right lung was involved in 56.8% of the cases and 58.2% of the controls (*p* = 0.875). When looking at the specific lobe involvement, there was no significant difference between cases and controls (*p* = 0.658).

### 3.3. Analysis of Unstandardized and Standardized Questionnaires

Table 3 displays the unstandardized questionnaire responses of the study cohort. Regarding the overall health status after the surgery, the mean score for the cases was 6.6, while for the controls, it was 7.8 (*p* = 0.016), suggesting that patients who developed bronchiectasis after surgery rated their health status lower than those who did not. In relation to symptom improvement, a significantly higher percentage of cases (27.3%) reported the worsening of their respiratory symptoms post-surgery compared to the controls (12.1%, *p* = 0.027). As for adherence to post-surgical care recommendations, 43.2% of cases reported compliance compared to 28.6% of the controls; however, this difference did not reach statistical significance. 

When evaluating smoking status, a lower percentage of cases (15.9%) reported a decrease in smoking frequency compared to the controls (31.9%, *p* = 0.049), suggesting more controls reduced their smoking post-surgery. The level of physical activity decreased significantly more in cases (47.7%) than in controls (19.8%) post-surgery (*p* < 0.001). In terms of the post-operative complications, 22.7% of cases reported experiencing complications or unexpected health issues post-surgery compared to 14.3% of controls (*p* = 0.221). Patients who developed bronchiectasis rated their mental/emotional well-being significantly lower post-surgery (mean 7.0) than the controls (mean 8.1) (*p* = 0.040). Lastly, cases were slightly less satisfied with the follow-up care they received (mean 7.3) than controls (mean 7.9); although, the difference was not statistically significant.

Table 4 presents the analysis of the World Health Organization Quality of Life Questionnaire—Brief Version (WHOQOL-BREF) at the six-month follow-up point. In this analysis, lower scores denoted a more negative quality of life. In the physical domain, the mean score was significantly lower in cases (14.0) than in controls (15.9), with a *p*-value of 0.043, implying that the cases reported a poorer physical quality of life than the controls. Similarly, the mean score in the mental domain was significantly lower for cases (11.5) compared to controls (13.0), with a *p*-value of 0.044, indicating that those who developed bronchiectasis had a worse mental quality of life than those who did not develop the condition.

**Figure 2:**
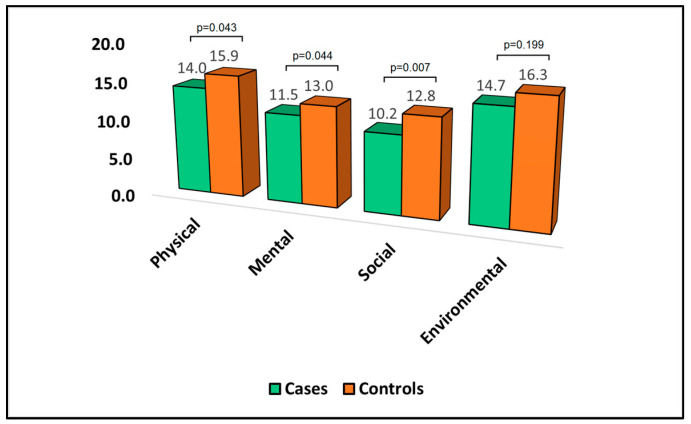
Analysis of WHOQOL-BREF questionnaire.

In the social domain, cases had a mean score of 10.2, which was significantly lower than the mean score for the controls (12.8), with a *p*-value of 0.007, suggesting a more negatively impacted social quality of life in cases, as presented in Figure 2. In contrast, in the environmental domain, the difference in the mean scores between cases (14.7) and controls (16.3) was not statistically significant, suggesting that there was no significant difference in environmental quality of life between the two groups.

**Figure 3:**
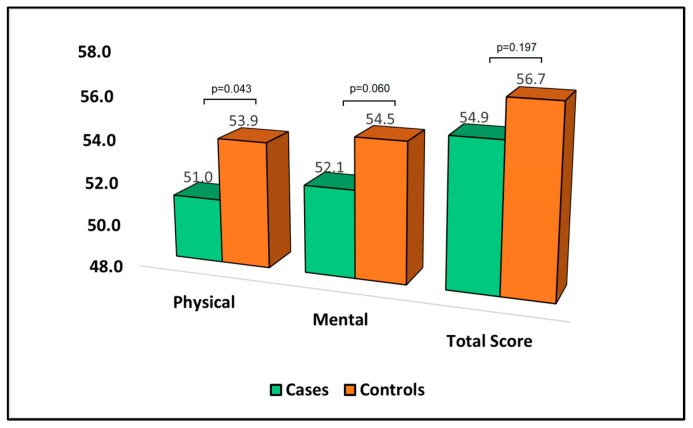
Analysis of SF-36 questionnaire.

Table 5 provides an analysis of the 36-Item Short Form Survey (SF-36) questionnaire at the six-month follow-up. The score for each domain of this survey was indirectly proportional to the degree of disability experienced by the patient. In the physical domain of the SF-36, the mean score was significantly lower for cases (51.0) than for controls (53.9. This suggests that patients who developed bronchiectasis after pulmonary resection experienced a higher degree of physical disability than those who did not develop bronchiectasis. The mean score in the mental domain was lower for cases (52.1) compared to controls (54.5); although, this difference did not reach statistical significance. This indicates that patients who developed bronchiectasis may have experienced a higher level of mental disability; however, the difference was not statistically significant. In terms of the total score, the mean for cases was 54.9 compared to 56.7 for controls, as presented in Figure 3.

Table 6 presents the analysis of the Hospital Anxiety and Depression Scale (HADS) questionnaire at the six-month follow-up. HADS is a measure where higher scores indicate more severe symptoms of anxiety and depression. The mean anxiety score was significantly higher in cases (7.52) than in controls (6.15), with a *p*-value of 0.037. This result suggests that patients who develop bronchiectasis after surgery experience more severe anxiety symptoms compared to patients who do not develop bronchiectasis.

**Figure 4:**
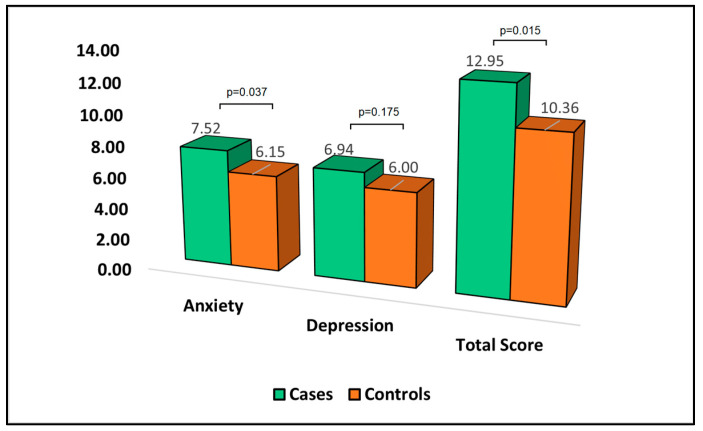
Analysis of HADS questionnaire.

In terms of depression, the mean score was higher in cases (6.94) than controls (6.00); however, this difference did not reach statistical significance. Although patients who developed bronchiectasis appeared to exhibit more severe depression symptoms, the difference was not statistically significant. The total HADS score, which sums the anxiety and depression scores, was significantly higher in cases (12.95) than in controls (10.36), with a *p*-value of 0.015, as described in Figure 4. This indicates that patients who develop bronchiectasis after surgery have a higher overall burden of anxiety and depression symptoms.

**Figure 5:**
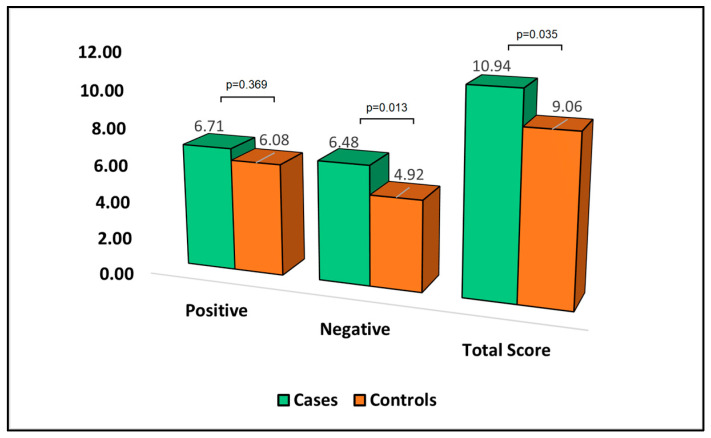
Analysis of PSS-10 questionnaire.

Table 7 reports the analysis of the Perceived Stress Scale (PSS-10) questionnaire at the six-month follow-up. The PSS-10 is a measure where higher scores indicate a greater degree of perceived stress. When it came to positive aspects of the questionnaire, the mean score for cases was 6.71, slightly higher than the control group with 6.08 (*p*-value = 0.369). In contrast, the negative dimension of perceived stress was significantly higher in cases (6.48) compared to controls (4.92), with a *p*-value of 0.013. This suggests that patients who develop bronchiectasis after surgery perceive their stress to be more negative than those who do not develop bronchiectasis. Moreover, the total PSS-10 score was significantly higher in cases (10.94) than controls (9.06), with a *p*-value of 0.035, as can be observed in Figure 5. This indicates that patients who develop bronchiectasis after surgery have a greater overall degree of perceived stress compared to those who do not develop bronchiectasis.

Age demonstrated a positive correlation with the SF-36 Physical (r = 0.366), suggesting that older patients tended to report better physical functioning. The SF-36 Physical score showed a strong positive correlation with the WHOQOL-BREF Physical score (r = 0.593), indicating that patients who scored higher on physical functioning also tended to report a better physical quality of life. It also had a significant correlation with the PSS-10 Negative score (r = 0.461), indicating that better physical functioning was associated with a higher degree of perceived negative stress.

**Figure 6:**
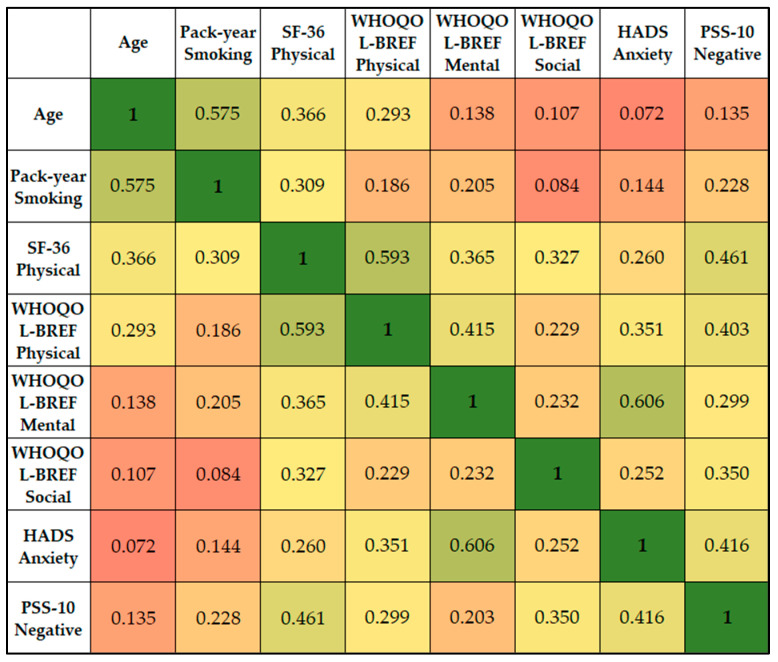
Correlation matrix between significant survey scores and patient’s condition.

The WHOQOL-BREF Physical score had a moderate correlation with HADS Anxiety (r = 0.351), indicating that a higher physical quality of life was associated with higher anxiety scores. The WHOQOL-BREF Mental score showed the strongest correlation with HADS Anxiety (r = 0.606), suggesting that greater mental/emotional well-being was associated with higher anxiety levels. The PSS-10 Negative score showed significant correlations with SF-36 Physical (r = 0.461), WHOQOL-BREF Physical (r = 0.403), WHOQOL-BREF Social (r = 0.350), and HADS Anxiety (r = 0.416), as presented in Figure 6. This indicates that a higher degree of perceived negative stress is associated with better physical functioning, higher physical and social quality of life, and higher anxiety levels.

## 4. Discussion

### 4.1. Literature Findings

The current study explored the health-related quality of life and stress-related disorders in patients who developed bronchiectasis after pulmonary resections. When comparing patient demographics and medical histories, there was no significant difference between the cases and controls. However, our findings reveal significant differences in the self-reported health status, symptom improvement, smoking behavior, and physical activity, with patients developing bronchiectasis indicating lower ratings and more negative outcomes postoperatively.

Patients developing bronchiectasis after lung resections reported a significantly poorer quality of life, evidenced by the lower scores in the physical and mental domains of WHOQOL-BREF. This finding aligns with the study by Guilemany et al. [24], which reported a decrease in the health-related quality of life in patients with bronchiectasis compared to the control patients. Patients with bronchiectasis also reported more severe anxiety symptoms, as measured by the Hospital Anxiety and Depression Scale (HADS), and higher overall perceived stress, as measured by the Perceived Stress Scale (PSS-10). These findings echo the results of Olveira et al. [25], who reported higher levels of anxiety and depression in bronchiectasis patients, suggesting a common mental health challenge for these patients.

As expected, older patients had a significantly higher pack-year smoking, which underscored the long-term health implications of tobacco use. This result is consistent with the previous research of Eisner et al. [26], who reported a significant association between the duration of smoking and the development of chronic respiratory conditions, including bronchiectasis. Moreover, our study further revealed a strong positive correlation between physical functioning (SF-36 Physical) and physical quality of life (WHOQOL-BREF Physical). This finding is in line with the study by Wilson et al. [27], which emphasized the influence of physical well-being on the overall quality of life in patients with respiratory conditions.

Interestingly, our data also show a significant correlation between higher physical quality of life and higher anxiety scores. This may seem counterintuitive, as one might expect better physical health to correlate with lower anxiety. However, this finding resonates with the ‘disability paradox’ suggested by Albrecht and Devlieger [28], where individuals with significant health challenges might perceive their quality of life differently than the general population. Although our research did not evaluate the correlation between physical assessment tests and quality of life in patients who developed bronchiectasis after pulmonary nodule resections, in a study involving almost 100 adult bronchiectasis patients with an average age of 69 years old, the investigators identified frequent exacerbations, extensive radiological disease, and a high bronchiectasis severity index (BSI) as the main factors contributing to poor quality of life [29]. The study noted that neither the underlying cause of bronchiectasis nor any comorbidities had an observable impact on the QoL. Exacerbations were related to the bacterial colonization of the bronchial tree, reduced forced expiratory volume in one second (FEV1), and more severe disease as shown by radiology. Interestingly, a higher BSI and a greater modified Medical Research Council (mMRC) dyspnea score were associated with lower physical quality of life scores. This research is useful in highlighting the intricate interplay between clinical severity measures and patient-reported quality of life measures in bronchiectasis.

The strong correlation between the WHOQOL-BREF Mental score and HADS Anxiety in our study supports the idea that mental/emotional well-being is strongly interlinked with anxiety levels, as has been highlighted in other research, such as a study by Ng et al. [30]. The association of higher perceived negative stress with better physical functioning and higher physical and social quality of life also underscores the complex relationship between physical health, mental well-being, and perceived stress in patients with bronchiectasis.

Moreover, we noted an intriguing paradox in our study that patients with bronchiectasis, despite experiencing more health concerns, reported a significantly lower frequency of smoking cessation compared to the control group. This observation is contrary to what might be expected given the well-established health risks of smoking, especially for those with existing respiratory conditions [31]. One possible explanation for this trend could be the influence of stress and anxiety, which are commonly seen in patients with bronchiectasis [32]. High levels of anxiety and stress could make it more challenging for these patients to quit smoking, as nicotine use has been linked to temporary stress relief [33]. Additionally, according to the self-medication hypothesis, individuals with high stress levels might use smoking as a maladaptive coping mechanism [34]. Future research should aim to explore these psychosocial dynamics in greater detail, potentially targeting stress and anxiety management as part of comprehensive cessation programs for this population.

Thus, the current study underscores the importance of considering both physical and mental health in the care of patients who develop bronchiectasis after a pulmonary resection. Nevertheless, even though we tried to exclude the possible causes of bronchiectasis prior to the 6-month follow-up, it was not possible to exclude the possibility of bronchiectasis existing before the surgical intervention and not being diagnosed. Future research should attempt to better control these factors and explore interventions to improve health-related quality of life, decrease anxiety and stress, and enhance self-management strategies in these patients. Moreover, the intriguing correlations found in this study warrant further exploration, to better understand the paradoxical relationships between physical and mental health in patients with bronchiectasis.

### 4.2. Study Limitations and Future Perspectives

This study, investigating the health-related quality of life and stress-related disorders in patients with bronchiectasis after pulmonary resections, presents several limitations that warrant consideration. Firstly, our study design was cross-sectional, which inherently limited our ability to establish causality. It only provided a snapshot of the relationships among the variables at a specific time, making it challenging to determine whether the observed associations represented cause–effect relationships. Secondly, the sample size was determined using a convenience sampling approach, which may have introduced selection bias and potentially limited the generalizability of the results. Our cohort of 44 patients with bronchiectasis after pulmonary resection was relatively small, which might have had an impact on the robustness of our findings. Additionally, our control group comprised patients who did not develop bronchiectasis following lung resection surgery, making our findings specific to this particular population and potentially not applicable to all patients with bronchiectasis. Moreover, the current study did not include in the analysis the extent and severity of bronchiectasis, which might contribute to worse HRQOL.

Additionally, while we excluded patients with significant comorbidities, the presence of minor or undiagnosed comorbidities could have impacted our results. Moreover, we did not account for the potential impact of different medications or treatments that participants might have been receiving, which could potentially confound our results. Finally, one of the limitations of the present study was the exclusion of patients with a malignant histology. While this was intentional to minimize confounding variables, we recognized the clinical importance of studying the HRQOL and stress outcomes in this subgroup, given that many lung resections occurred due to malignancies. The future research aims to assess these parameters in patients with malignant lung nodules to provide a comprehensive understanding of the impacts of lung resections on HRQOL and stress-related disorders. These limitations notwithstanding, we believe our findings provide valuable insights into the relationship between bronchiectasis post-lung resection, quality of life, and stress-related disorders. However, further longitudinal studies with larger and more diverse patient samples, including various comorbidities, are required to confirm and extend our findings.

## 5. Conclusions

In conclusion, our study findings suggest that patients who developed bronchiectasis post-pulmonary resection surgery experienced a lower health-related quality of life, higher levels of perceived stress, and higher symptoms of anxiety compared to those who did not develop bronchiectasis. Despite no significant differences in the demographic and clinical characteristics between the two groups, those developing bronchiectasis rated their overall health status lower and reported a higher incidence of deteriorating respiratory symptoms. Physical, mental, and social domains of life quality were negatively impacted as assessed by WHOQOL-BREF, while physical disability was higher as per SF-36 scoring. Anxiety levels were significantly higher in these patients, and overall burden of anxiety and depression was more pronounced as per HADS analysis. These findings highlight the need for more comprehensive post-surgical care and mental health support for patients undergoing lung resection surgery, particularly those who develop bronchiectasis. Moreover, detailed imaging studies and respiratory function assessments can be performed after nodule resection to evaluate the risk of bronchiectasis development. Further research is required to understand the underlying mechanisms and develop strategies to mitigate the negative impact of surgery on quality of life and mental health in these patients.

## Figures and Tables

**Table 1:** Patients’ demographics and medical history.

Patients’ Background	Cases (*n* = 44)	Controls (*n* = 91)	*p*-Value
Age (mean ± SD)	56.2 ± 7.8	55.6 ± 7.1	0.656
Age range	42–66	40–65	–
Gender (men, %)	29 (65.9%)	64 (70.3%)	0.603
Area of residence (urban, %)	21 (47.7%)	42 (46.2%)	0.863
Smoking status (yes, %)	17 (38.6%)	30 (33.0%)	0.516
Pack-year smoking (median, IQR)	24.5 (16.0–31.5)	21.0 (15.5–33.0)	0.219
Prior exposure to respiratory hazards (yes, %)	9 (20.5%)	14 (15.4%)	0.462
Obesity (yes, %)	16 (36.4%)	22 (24.2%)	0.139
CCI > 3 (*n*, %)	10 (22.7%)	16 (17.6%)	0.477

SD—standard deviation; IQR—interquartile range; CCI—Charlson comorbidity index; cases—developed bronchiectasis after surgery; controls—did not develop bronchiectasis.

**Table 2:** Clinical characteristics and investigations in the study cohort.

Variables	Cases (*n* = 44)	Controls (*n* = 91)	*p*-Value
FEV1% (mean ± SD)	80.3 ± 8.7	81.8 ± 7.5	0.303
EF% (mean ± SD)	57.0 ± 3.3	56.4 ± 3.9	0.380
Benign histology (yes, %) *	44 (100%)	91 (100%)	-
Localization			0.875
Left lung (*n*, %)	19 (43.2%)	13 (41.8%)	
Right lung (*n*, %)	25 (56.8%)	16 (58.2%)	
Lobe involved			0.658
Left upper lobe	11 (25.0%)	23 (25.3%)	
Left lower lobe	6 (13.6%)	10 (11.0%)	
Right upper lobe	10 (22.7%)	24 (26.4%)	
Right middle lobe	4 (9.1%)	15 (16.5%)	
Right lower lobe	13 (29.5%)	19 (20.9%)	

SD—standard deviation; *—diagnosed after resection surgery; cases—developed bronchiectasis after surgery; controls—did not develop bronchiectasis.

**Table 3:** Unstandardized questionnaire responses.

Variables	Cases (*n* = 44)	Controls (*n* = 91)	*p*-Value
Post-Operative Health Status: On a scale of 1 to 10, how would you rate your overall health status after the surgery?—Mean (SD)	6.6± 3.0	7.8 ± 2.5	0.016
Symptom Improvement: Have your respiratory symptoms improved, worsened, or stayed the same after the surgery?—Worsened (%)	12 (27.3%)	11 (12.1%)	0.027
Adherence to Recommendations: Did you follow all the post-surgical care recommendations provided by the healthcare team?—Yes (%)	19 (43.2%)	26 (28.6%)	0.091
Smoking Status: Are you currently smoking? If yes, has the frequency of smoking increased, decreased, or remained the same after surgery?—Decreased (%)	17 (15.9%)	29 (31.9%)	0.049
Physical Activity: Has your level of physical activity changed after the surgery? Has it increased, decreased, or stayed the same?—Decreased (%)	21 (47.7%)	18 (19.8%)	<0.001
Post-Operative Complications: Did you experience any complications or unexpected health issues after the surgery?—Yes (%)	10 (22.7%)	13 (14.3%)	0.221
Mental Well-Being: On a scale of 1 to 10, how would you rate your mental/emotional well-being after the surgery?—Mean (SD)	7.0 ± 3.6	8.1 ± 2.5	0.040
Follow-Up Care Satisfaction: On a scale of 1 to 10, how satisfied are you with the follow-up care you received after the surgery?—Mean (SD)	7.3± 2.9	7.9 ± 3.4	0.316

SD—standard deviation; cases—developed bronchiectasis after surgery; controls—did not develop bronchiectasis.

**Table 4:** Analysis of WHOQOL-BREF questionnaire at the six-month follow-up.

Domains	Cases (*n* = 44)	Controls (*n* = 91)	*p*-Value
Physical	14.0 ± 4.8	15.9 ± 5.2	0.043
Mental	11.5 ± 3.1	13.0 ± 4.4	0.044
Social	10.2 ± 4.6	12.8 ± 5.5	0.007
Environmental	14.7 ± 6.2	16.3 ± 7.0	0.199

WHOQOL-BREF—World Health Organization Quality of Life Questionnaire—Brief Version; lower scores indicate a more negative quality of life; cases—developed bronchiectasis after surgery; controls—did not develop bronchiectasis.

**Table 5:** Analysis of SF-36 questionnaire at the six-month follow-up.

SF-36	Cases (*n* = 44)	Controls (*n* = 91)	*p*-Value
Physical	51.0 ± 7.2	53.9 ± 8.0	0.043
Mental	52.1 ± 8.0	54.5 ± 6.3	0.060
Total score	54.9 ± 7.9	56.7 ± 7.4	0.197

SF-36—36-Item Short Form Survey; the score for each domain is indirectly proportional to the degree of disability experienced by the patient; cases—developed bronchiectasis after surgery; controls—did not develop bronchiectasis.

**Table 6:** Analysis of HADS questionnaire at the six-month follow-up.

HADS	Cases (*n* = 44)	Controls (*n* = 91)	*p*-Value
Anxiety	7.52 ± 4.06	6.15 ± 3.28	0.037
Depression	6.94 ± 3.14	6.00 ± 4.02	0.175
Total score	12.95 ± 6.28	10.36 ± 5.44	0.015

HADS—Hospital Anxiety and Depression Scale; a higher score describes higher negative symptoms of anxiety and depression; cases—developed bronchiectasis after surgery; controls—did not develop bronchiectasis.

**Table 7:** Analysis of the PSS-10 questionnaire at the six-month follow-up.

Domain (Mean ± SD)	Cases (*n* = 44)	Controls (*n* = 91)	*p*-Value
Positive	6.71 ± 3.62	6.08 ± 3.90	0.369
Negative	6.48 ± 3.15	4.92 ± 3.49	0.013
Total score	10.94 ± 4.18	9.06 ± 5.11	0.035

PSS-10—The Perceived Stress Scale; a higher score suggests a higher degree of perceived stress; cases—developed bronchiectasis after surgery; controls—did not develop bronchiectasis.

## Data Availability

Data available on request.
